# Detection and Identification of Avian Reovirus in Young Geese (*Anser anser domestica*) in Poland

**DOI:** 10.3390/ani12233346

**Published:** 2022-11-29

**Authors:** Tomasz Nowak, Adam Kwiecinski, Piotr Kwiecinski, Grzegorz Tomczyk, Karolina Wodz

**Affiliations:** 1 Department of Molecular Biology, Vet-Lab Brudzew, 62-720 Brudzew, Poland; 2 Department of Poultry Diseases, National Veterinary Research Institute, 24-100 Puławy, Poland

**Keywords:** goose, reovirus, arthritis

## Abstract

**Simple Summary:**

Infection with goose reovirus (GRV) can cause serious economic losses in the goose breeding industry. In this study, we determined infectious agents associated with arthritis and the generalized infection of a flock of young geese in Poland, using molecular biology, histopathology, and virus isolation methods. The disease caused by GRV has no cure or effective vaccine. The early detection of its pathogen is basic to prevent of secondary bacterial and fungal infections.

**Abstract:**

Avian reovirus (ARV) is a cause of infections of broiler and turkey flocks, as well as waterfowl birds. This case report describes a reovirus detection in a fattening goose flock. GRV-infected geese suffer from severe arthritis, tenosynovitis, pericarditis, depressed growth, or runting-stunting syndrome (RSS), malabsorption syndrome, and respiratory and enteric diseases. GRV (goose reovirus) caused pathological lesions in various organs and joints, especially in the liver and spleen. GRV infection causes splenic necrosis, which induces immunosuppression, predisposing geese to infection with other pathogens, which could worsen the disease and lead to death. Our results showed that GRV was detected via RT-PCR and isolated in SPF (Specific Pathogen Free) embryos. This is the first report of the involvement of reovirus in arthritis, and the generalized infection of young geese in Poland, resulting in pathological changes in internal organs and sudden death. This study also provides new information about the GRV, a disease that is little known and underestimated.

## 1. Introduction

Reovirus infections of chicken and turkey are common, and most commercial poultry flocks probably become infected during the life of the flock. Avian reoviruses (ARVs) are widespread worldwide and affect various avian species [1], including chickens [2,3], turkeys [3], ducks [4,5,6], psittacine birds [7], pigeons, and other wild birds [8]. Moreover, novel duck reovirus (NDRV) causes spleen necrosis and swelling, and it is associated with high mortality in Pekin ducklings [9,10,11,12]. Thus, reovirosis mainly affects commercially grown Muscovy ducklings, and has commercial importance in areas with intensive Muscovy duck production [9,10,11,12]. However, very little is still known about reovirus infections in geese [6,13].

Avian reovirus of Muscovy ducks was first described in South Africa in 1950 [14], and then in France in 1972 [15], where the virus was isolated. In 1997, Muscovy duck breeding regions in China were affected by an outbreak of duck reovirus (DRV) disease, which resulted in white spots on the liver, after its death, due to infection [16]. In 2011, an outbreak of duck reovirus (DRV) was notified in Pekin ducks in China, and large necrotic foci in the spleens were described [17]. In 2003, Palya et al. [13] detected antibodies against goose reovirus (GRV) in the serum and in different organs of the affected geese. The authors also reported that GRV causes arthritis, spleen inflammation in the acute phase, pericarditis, arthritis, and/or tenosynovitis in the subacute or chronic phase [13], and in adult geese, GRV is an agent of infection [13].

ARV belongs to the genus *Orthoreovirus*, family *Reoviridae* [18]. It is a nonenveloped virus with double-stranded RNA (dsRNA), consisting of 10 linear segments encoding eight structural and three or four [19,20] non-structural viral proteins in the European GRV D20/99 strain. The complete genome consists of 23,420 nucleotide base pairs (bp), including segments ranging from 1191 bp (S4) to 3959 bp (L1). The genetic material is surrounded by an icosahedral inner and outer capsid consisting of eight structural proteins. To date, a few complete genomic sequences of chicken ARV strains [21,22], turkey arthritis reovirus (TARV) [23], muscovy duck reovirus (DRV) [24,25], and goose [26] and gosling ARVs [27] from China have been reported. Several differences between chicken ARV and duck reovirus (DRV) have been noted, including antigenicities, and host and pathogenic features [24]. Although duck and goose ARVs share high sequence similarity of the S1 segment, which mediates the attachment to the host cells [28], as well as biological and molecular features [13], they should not be classified as one species. Phylogenetic analysis of the σC gene indicates that GRV belongs to neither the chicken nor the duck reovirus cluster [25]. Thus, GRV could be recognized as a new species within the ARV group. Description of a goose ARV infection will help to better understand this poorly known virus, and it may also be useful for taking into consideration the improvements of preventive measures (such as designing better diagnostic tools and developing effective vaccines).

The aim of this study was to determine infectious agents associated with arthritis and the generalized infection of a flock of 15,000 young geese in Poland, using molecular biology and virus isolation methods to detect the viruses associated with the infection of geese. Moreover, it aims at the improvement of the PCR detection of ARV, GRV, and NDRV.

## 2. Materials and Methods

### 2.1. Case Report

The disease was observed in a fattening goose flock of 15,000 individuals. The flock inhabited one house owned by a single farmer in western Poland. The flock was given ready-made feed. The earliest onset of the symptoms related to a suspected ARV infection was observed on the 20th day after hatching, with the outbreak occurring in the third week of life. The clinical signs in the acute phase included general malaise, diarrhea, and locomotive problems. The hock, metatarsal and digital joints, and the flexor tendon and synovial bursae were swollen. The symptoms lasted two weeks after the onset. The flock was given symptomatic treatment with kidney and liver supporting supplements with ADE vitamin complex over a period of three weeks.

#### Gross Pathology, Bacteriology and Sample Collection

Complete necropsies were performed on 15 birds in the acute phase of the disease, 8 of which were dead and 7 euthanized through decapitation. Samples of liver, spleen, lung, kidney, intestine, brain, and joints were routinely cultured using commercial aerobic growth media (Columbia Agar with 5% sheep blood, Columbia Naladixic Acid Agar with 5% sheep blood, MacConkey agar, OXOID, Hampshire, UK), anaerobic growth media (liquid Schaedler medium with hemine and vitamin K, solid Schaedler agar with sheep blood and vitamin K, both OXOID, Hampshire, UK) and mycologic growth media (Sabouraud Glucose Agar with Gentamicin and Chloramphenicol, OXOID, Hampshire, UK), due to a suspicion of bacteriological (*E. coli*, *P. multocida*, *R. anatipestifer*, *E. rhusipoathiae*, *S. gallolyticus*, *C. perfringens)*, and fungal *(A. fumigatus*) infection.

Samples from liver, spleen, kidney, intestine, cloacal bursa and sciatic nerve were fixed in formalin 1:10 (vol:vol) (Bio Optica, Milan, Italy) for further histopathological studies. Samples of liver, spleen, joints, and other organs (heart, kidney, tendon, tendon sheath, lungs, brain, and intestines) were collected and frozen (−20 °C) to further molecular study and virus isolation in chicken SPF embryos. All analysis were performed separately for each organ and for each examined birds.

### 2.2. Histopathology

Fixed samples were processed according to routine histopathological practice using automated tissue processor (Tissue-Tek VIP 6AI, Sakura, Japan), with a range of fixers embedded in paraffin wax (Tissue-Tek TEC, Sakura, Japan), cut into thin sections of 4 μm thickness (Accu-Cut SRM, Sakura, Japan), and stained with hematoxylin and eosin (Stamar, Dąbrowa Górnicza Poland). The slides were examined for lesions using a light microscope (Olympus CX43). Microphotographs of all examined organs were subjected to computer-assisted image analysis, a computer coupled to an Olympus BX53 optical microscope (Olympus, Shinjuku, Tokyo, Japan) and using Olympus cell Sens Entry imaging software (Olympus Soft Imaging Solution GmbH, Munster, Germany) to increase contrast and sharpness details.

### 2.3. Virus Detection

#### 2.3.1. ARV Detection

The swabs (FLOQSwabs, Copan, Italy) with homogenized organs were immediately placed in 600 µL R9F buffer and vortexed, according to the manual (A&A Biotechnology, Gdańsk, Poland). RNA was extracted from swab using Viral DNA/RNA kit (A&A Biotechnology, Gdańsk, Poland), according to the manufacturer’s instructions. Viral RNA was reverse transcribed to cDNA with a Maxima First Strand cDNA Synthesis Kit for RT-qPCR (Thermo Fisher Scientific, Waltham, MA, USA). The same amount of RNA was used for tests order to normalize the amount of RNA in individual samples.

For the detection of avian orthoreovirus, PCR was performed using DreamTaq Green PCR Master Mix (2×) (Thermo Fisher Scientific, Waltham, MA, USA), 1 μL of each 10 μM primers (ARVF 5′-GGA GGT ACG TGT GCC AAA C-3′ and ARVR 5′-GTG CCT ACC AAC CAC ACT C-3′), located in segment S3, sigma-B protein (Genomed, Warsaw, Poland), and the GeneAmp PCR System 2700 (Thermo Fisher Scientific, Waltham, MA, USA). The reaction conditions were as follows: initial denaturation at 95 °C for 3 min, followed by 35 cycles of denaturation at 95 °C for 30 s, annealing at 54 °C for 30 s, and extension at 72 °C for 1 min, with a final extension for 7 min at 72 °C [13]. The PCR products were visualized using a 2% agarose (Thermo Fisher Scientific, Waltham, MA, USA) gel with ethidium bromide under UV light conditions (UVP GelSolo, Analtyic Yena, Jena, Germany).

In parallel, to confirm the PCR result, real-time RT-PCR to detect the RNA of avian orthoreovirus Nonstructural Protein σ was performed using an Avian Orthoreovirus Advanced kit (Genesig, Primerdesign, Chandler’s Ford, UK) and oasig lyophilized OneStep RT-qPCR Master Mix (Genesig, Primerdesign, Chandler’s Ford, UK) according to the manufacturer’s instructions, and Applied Biosystems 7500 Fast Real-Time PCR System (Thermo Fisher Scientific, Waltham, MA, USA). The same amount of RNA was used for tests order to normalize the amount of RNA in individual samples. Water was run as the negative control, and a commercial positive control from the Genesig kit was run as the positive control.

#### 2.3.2. GRV and Geese/Duck Virus Detection

For the detection of the Goose reovirus σC RNA, PCR was performed using DreamTaq Green PCR Master Mix (2×) (Thermo Fisher Scientific, Waltham, MA, USA) and 1 μL of each 10 μM primers (GRV-F 5′-TGA GAC GCC TGA CTA CGA TT-3′ and GRV-R5′- ATG CTT GGA GTG AGA CGA CT-3′) [16] (Genomed, Warsaw, Poland), and the GeneAmp PCR System 2700 (Thermo Fisher Scientific, Waltham USA). The reaction conditions were as follows: initial denaturation at 95 °C for 3 min, followed by 35 cycles of denaturation at 95 °C for 30 s, annealing at 54 °C for 30 s, and extension at 72 °C for 1 min, with a final extension for 7 min at 72 °C. PCR products were visualized under a UV light (UVP GelSolo, Analtyic Yena, Jena, Germany) following electrophoresis through 2% ethidium bromide stained agarose gels (Thermo Fisher Scientific, Waltham, MA, USA).

For the detection of NDRV, TaqMan-based, real-time RT-PCR was performed using a oasig lyophilized OneStep RT-qPCR Master Mix (Genesig, Primerdesign, Chandler’s Ford, UK), primers targeting the NDRV S3 protein gene (NDRV_F 5′-GGTTGGAAGATGATAGATG-3′, NDRV R 87 5′-GGCAGTGGTTACTAACATC-3′), the NDRV- and TaqMan-based probe (FAM-5′ CACACTCTTCCTGACTGTCTGGT-3′-BHQ1) [10], and an internal control primer/probe mix (Genesig, Primerdesign, Chandler’s Ford, UK), according to the manufacturer’s instructions, and an Applied Biosystems 7500 Fast Real-Time PCR System (Thermo Fisher Scientific, Waltham, MA, USA).

The detection of goose hemorrhagic polyomavirus (HNEG) and goose and duck parvovirus (GPV/MDPV) was investigated via real-time PCR with commercial kits (Goose Polyomavirus and MDPV/GPV Typing, Kylt, Hoeltinghausen, Germany, respectively), according to the manufacturer’s instructions, and an Applied Biosystems 7500 Fast Real-Time PCR System. The detection of goose circovirus (GCV) and duck circovirus (DuCV) was performed via PCR with a commercial end-point PCR Duck Circovirus kit (Kylt, Hoeltinghausen, Germany), and the GeneAmp PCR System 2700.

### 2.4. Virus Isolation in Chicken SPF Embryos

Virus isolation in chicken SPF embryos, detection and confirmation of presence of ARV was performed in The National Veterinary Research Institute, Poland. The supernatants of organ homogenates and fluid from joints were diluted and filtered through 0.45 µm syringe filters. The filtered suspension was injected into the yolk sac of 5–7-day-old specific pathogen free (SPF) chicken embryos (Valo, Lohman Tierzucht, Cuxhaven, Germany), at 0.2 mL/embryo [8,29]. The embryos were candled daily for seven days. The amnio-allantoic fluids, membranes, livers, and spleens of the infected embryos were collected for further collected for further ARV detection using PCR.

## 3. Results

### 3.1. Clinical Observations

Approximately 50% of the flock expressed clinical signs of the disease. Morbidity was 47% and mortality 15% The clinical signs in the acute phase were locomotory problems, lameness, and sudden death in 2nd and 3rd day after first signs. The clinical signs noted in the outbreak were depression, reluctance to move, increased water consumption but anorexia, nasal discharge and conjunctivitis, dyspnea, occasionally greyish watery diarrhea, the peeling of skin from feet and bill, leg weakness, and rare neck tremor or paresis. The hock and metatarsal or digital joints, as well as the gastrocnemius and digital flexor tendons were markedly swollen. Birds that survived the acute phase of the disease developed chronic lameness, were markedly stunted in growth, and the weight gain was significantly inhibited, below 6.3–6.5 kg, distributed unevenly. In birds which survived the acute phase, myositis was noted in clinical examination.

### 3.2. Gross Pathology and Bacteriology

**Figure 1:**
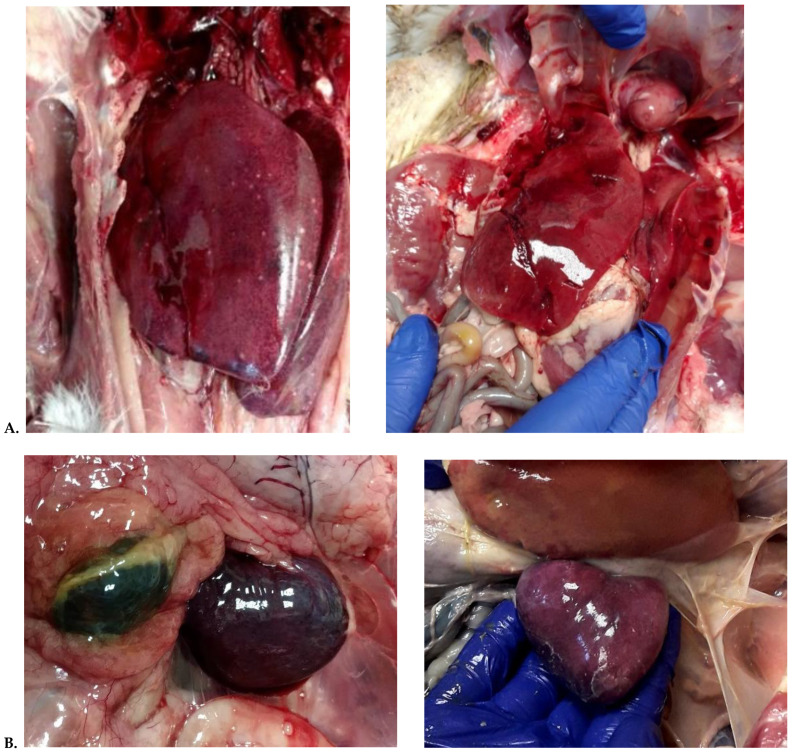

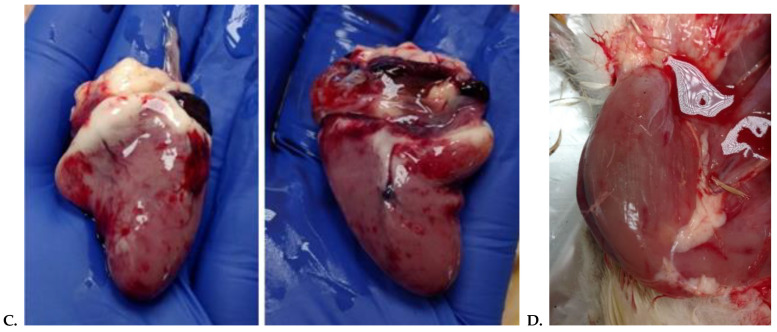
(**A**) disseminated, white necrotic spots in an enlarged and congested liver; (**B**) enlarged and congested spleen with clear foci suggestive of necrosis; (**C**) epicardial multifocal to coalescing haemorrhage on the surface of the heart muscle; (**D**) Leg muscle haemorrhages and subcutaneous oedema from a GRV infected goose.

Twelve of the fifteen examined birds showed pathological lesions. Hepatomegaly was observed, with multiple disseminated, greyish-white, slightly elevated pin-head necrotic foci and in most birds (Figure 1A). Liver was friable and congested. Spleen was mottled, dark, and enlarged amongst all examined geese (Figure 1B).

Kidneys and pancreas were swollen and pale. Less frequently, hemorrhages on the surface of the myocardium (Figure 1C) and hemorrhagic enteritis were observed in all areas of the intestine and caeca. Congestion of the lung and occasionally subcutaneous and leg muscle hemorrhages were observed (Figure 1D). Additionally, the presence of mucus in the upper respiratory tract, fibrinous airsacculitis, and serous exudation in the abdomen were observed. Sero-fibrinous epicarditis and pericarditis, and arthritis and tenosynovitis during the acute and chronic phase of the disease were observed. Additionally, the synovial bursae were markedly swollen. Large hemorrhages in the region of the gastrocnemius flexor tendon and the surrounding tissues as a consequence of the rupture of the tendon were observed in the chronic phase of the disease.

The presence of *E. coli* was noticed in bacteriology analysis for bacteria only in the liver and spleen in 3 of 15 examined birds as a secondary infection. No other bacteria or fungi which might have accounted for the lesions were cultured, what allowed for the exclusion of bacteria and fungi as the causative agents of the observed hemorrhagic and necrotic lesions in the examined organs.

### 3.3. Histopathology

**Figure 2:**
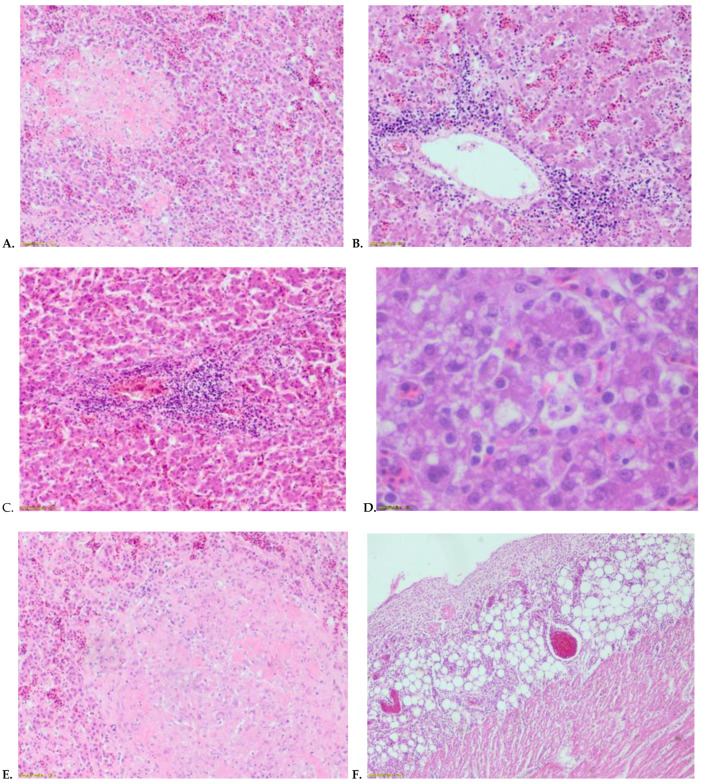

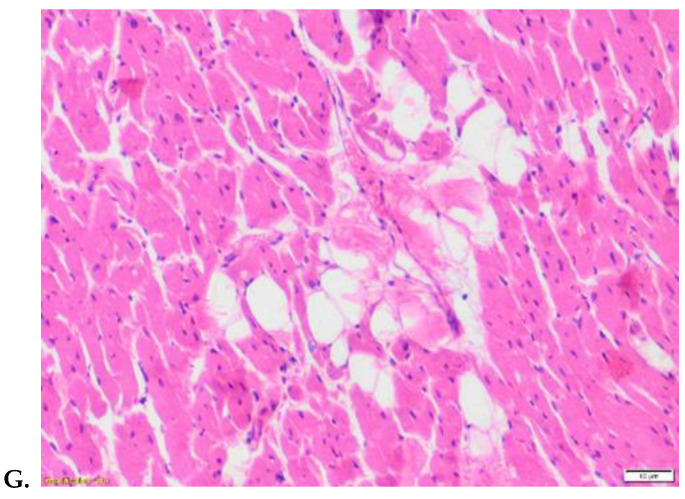
Liver, (**A**) focal hepatic necrosis, (**B**) perivescular lymphoplasmocytes and mecrophages infiltration, (**C**) perivescular inflammatory cells infiltrares, (**D**) vacuolar degeneration of hepatocytes. (**E**) Spleen, necrotic areas infiltrated by macrophages and plasma cells. Heart (**F**) expansion of epicardium and infiltration by inflammatory cells, extension of cells infiltrate to myocardium, subepicardial myocarditis, (**G**) vacuolization of cardiac myocytes. Hematoxylin and eosin stain, magnification ×40.

Miliary foci of vacuolated, necrotic hepatocytes, and focal necrotic areas which were infiltrated by lymphocytes, macrophages, and a few plasma cells in the liver were found (Figure 2A–D). In the spleen focal necrosis, the depletion of lymphoid follicles was observed. Additionally, splenic tissues were infiltrated by macrophages and plasma cells, and occasionally fibrin deposits (Figure 2E). The cardiac epicardium was observed by expansion, and the infiltration of inflammatory cells, which extended to the myocardium and caused sub-epicardial myocarditis. There was an area of myocardial necrosis and degeneration, with a marked infiltration of lymphocytes mixed with plasma cells and macrophages (Figure 2F,G). In tendons, acute tenosynovitis with the exudative inflammation of synovial sheath, the hyperplasia of synovial epithelial cells, fibrinous exudate in the synovial cavity, and mild infiltration of inflammatory cells into tendons were observed (data not shown). In the kidney, tubular degeneration and necrosis was seen, with marked inflammatory infiltrates around the blood vessels. Plasma cellular infiltrates were also found in the lamina propria of the intestine, along with crypts hyperplasia, cytoplasmic vacuolation, and the necrosis of enterocytes. No considerable lesions were observed in sciatic nerves; however, several lymphoid follicles of the bursa of Fabricius, both in the cortex and in the medulla, were depleted by the lymphocytes.

### 3.4. RT-PCR

**Figure 3:**
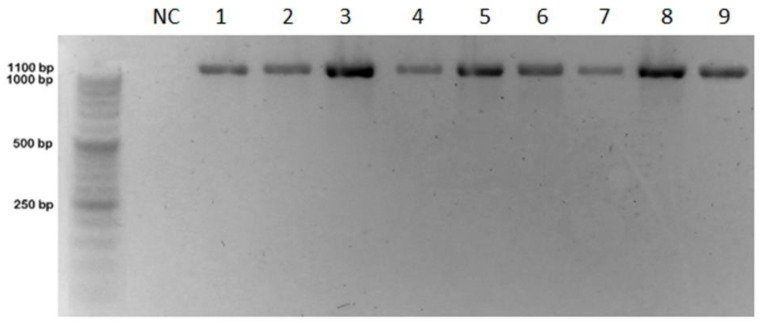
Detection avian reovirus (ARV) by RT-PCR; from left DNA Marker 2+ (100–1000 bp, A&A, Gdańsk, Poland), NC—negative control, 1—liver, 2—spleen, 3—bursa of Fabricius 4—joints, 5—tendon, 6—tendon sheath, 7—heart, 8—kidney, 9—intestines; PCR product size 1100 bp.

**Figure 4:**
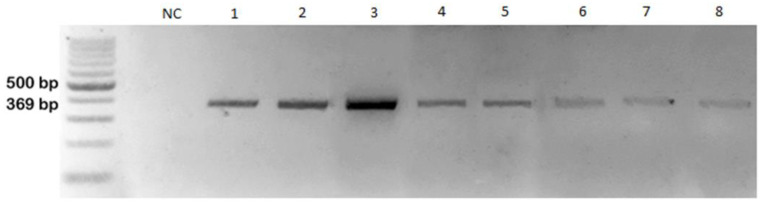
Detection of Goose Reovirus (GRV) by RT-PCR; from left DNA Marker 1 (A&A, Poland, 100–1000 bp), NC—negative control, 1—liver, 2—spleen, 3—joints, 4—tendon, 5—tendon sheath, 6—heart, 7—kidney, 8—intestines; product size 369 bp.

**Figure 5:**
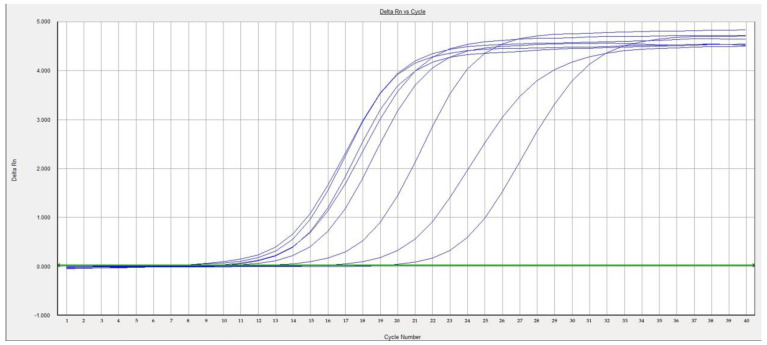
Detection of avian orthoreovirus via real-time RT-PCR.

ARV was detected, via RT-PCR and real-time RT-PCR, in liver, spleen, joints, tendon, tendon sheath, and other organs (bursa of Fabricius, heart, kidney, and intestines) in each individual bird. The 1100 bp PCR product was amplified using primers specific for avian orthoreovirus S3 segment (Figure 3) and 369 bp using primers specific for GRV σC protein (Figure 4). Real-time RT-PCR for the detection of avian reovirus (ARV) showed the presence of fluorescent curves at the cycle threshold values (Ct) ranging from 11 to 20 (Figure 5). Samples from all 15 geese were positive when using both methods. The results for the detection of NDRV were negative. To exclude other geese viral infections, real-time PCR and PCR methods to detect hemorrhagic polyomavirus (GHPV), goose and duck Parvovirus (GPV/MDPV), and goose circovirus and duck circovirus (GCV/DuCV) were conducted. The analysis did not show the presence of any of these viruses.

### 3.5. Virus Isolation

The virus was isolated from clarified organ homogenates and fluid from the joints of infected geese generated by three passages into the yolk sacs of 5–7-day-old specific-pathogen-free (SPF) chicken embryos. All embryos exhibited single necrotic lesions under the capsule of the liver. The mortality of the infected SPF embryos caused by ARV was low. ARV was detected in amino-allantoic fluids, membranes, livers, and spleens of infected embryos via RT-PCR.

## 4. Discussion

Geese meat and down are important products exported from Poland. The productivity of the goose industry may be affected by several infectious agents, including viral (HNEG, GPV/MDPV, and GCV/DuCV,) and bacterial (*Erysipelothrix rhusiopathiae*, *Pasteurella multocida*, *Escherichia coli*, and *Streptococcus gallolyticus*). GRV can also occur as a co-infection with other viruses and bacteria, making the GRV infection more severe [16].

Most of the reoviruses isolated from poultry seem to be non-pathogenic. However, pathogenic ARV has been associated with a number of different diseases. Previously, reovirus infections were reported mainly in chicken and ducks.

To date, a relatively small amount of data related to reoviruses infection in geese has been available.

Reovirus infection was reported in duck [5,6,25] and geese in many provinces of China [26,27], and severe reovirus infections, characterized by nephritis, hepatitis, and splenitis were reported in goslings in Shandong province in China in 2016 [30]. Similarly DRV infection is common in many provinces of China [9,10,11,12]. A study from Hungary characterized reovirus infection in geese (*Anser anser domestica*) as locomotor disorder and lameness accompanied by splenitis, hepatitis, epicarditis, arthritis, and tenosynovitis with high morbidity [13], and monitored the disease during the following years. To date, the epidemiology of GRV in Poland is obscure.

This case is a first description of generalized infection and GRV-born arthritis in young geese in Poland. Gross lesions of this disease indicated cell degeneration, swelling, and necrosis of the liver and spleen (Figure 1). Histopathological analysis also indicated cell degeneration and necrosis in both the liver and spleen (Figure 2).

A previous report on the occurrence of a novel duck reovirus (NDRV) from China shows the emergence of a duck reovirus causing 40% mortality in birds of various ages [17]. In our case report we observed similar mortality and clinical symptoms specific for reovirus infection in a fattening goose flock. Amongst the clinical signs, there were locomotory problems and lameness, indicating possible viral arthritis caused by avian reovirus (ARV). The pathological changes in liver and spleen, and sudden death, indicate possible novel duck reovirus (NDRV) infection. Moreover, to exclude other viral (HNEG, GCV/DuCV, or GPV/MDPV, NDRV) or bacterial infections (*E. coli*, *P. multocida*, *R. anatipestifer*, *E. rhusipoathiae*, or *S. gallolyticus*), real-time PCR, PCR tests, and bacteriological examination were conducted. The analysis did not show the presence of any of these viruses. The presence of *E. coli* was noticed in bacteriology analysis only in the liver and spleen, in 3 of 15 examined birds as a secondary infection. The results for the detection of NDRV were also negative.

ARV was isolated from infected geese using specific pathogen free (SPF) chicken embryos, and was detected in the amino-allantoic fluids, membranes, livers, and spleens of infected embryos via RT-PCR.

The ARV causes splenic necrosis and immune compromise, which favors bacteria such as *E. coli*, *S. aureus*, or *E. rhusiopatiae*. In ducks, chicken and turkey reovirus replicated in the bursa of Fabricius, which might induce a transient and possibly permanent state of immunosuppression in infected birds [31]. However, in our case, the fact that the virus was detected in the spleen, liver, and kidney of infected geese suggests that GRV was invasive for these internal organs, probably affecting the humoral immune response. Moreover, several lymphoid follicles of the bursa of Fabricius, both in the *cortex* and in the *medulla*, were depleted by the lymphocytes.

Thus, infection with goose reovirus (GRV) can cause great economic losses in the geese industry. Moreover, vertically transmitted GRV causes additional economic losses due to the mortality of the affected gosling, even though infected breeding geese do not show clinical signs. The earliest onset of the disease of young goslings is between 5 and 21 days of age, but GRV induced clinical symptoms mainly at 2–3 weeks of age [16]. The disease course lasted 3–6 weeks [13], but it may persist until 7 to 10 weeks of age [30]. In older birds, GRV replicates in the gastrointestinal tract, but without general symptoms.The main targets of the GVR are the spleen and liver, although the virus can infect the brain and joints. Morbidity ranges from 10 to 60%, and is always higher in young birds (2–3 weeks of age), while mortality varies from 2 to 20% in the same age.

The most effective way to prevent the disease is vaccination, similarly to the cases of reovirosis in hens (vaccine strain ARV-S1133). To date, vaccines for GRV are not available. In our case report, the confirmation of reovirus infection is based on laboratory analysis such as RT-PCR, real-time RT-PCR, and virus isolation.

A few limitations of the present study require consideration. First, one flock was tested, and thus, further studies in a larger group of flocks are required. Secondly, we did not sequence sigma C.

The strengths of this case report provide clinically useful data from gross pathology and histopathology. Additionally, the data collected during this examination correspond to that reported in single available studies.

The virus-induced damage of the immune organs implies that GRV infection may serve as a triggering or concurrent agent in the development of geese secondary viral or bacterial diseases. The routine detection of reovirus antibodies in sera, using the ELISA method, is not available. The replication of GRV in chicken embryos is an expensive, non-routine diagnostic method, and it is performed only in specialized laboratories. Therefore, molecular biology techniques, and isolation from the various organs and joints of infected geese might be valuable for the development of routine diagnostic tests for GRV and ARV infections.

## 5. Conclusions

The occurrence of novel viruses in poultry and waterfowl husbandry remains one of the most serious epizootic and economic problems to date. We may thus conclude that this is the first molecular study in Poland, to highlight the involvement of reovirus in arthritis and the generalized infection of young geese, resulting in changes in internal organs and sudden death. The primers used in our case reports allow for the effective detection of RNA in the internal organs of infected geese. The presented results confirm the utility of PCR methods in the diagnosis of arthritis and the generalized infection of geese. Moreover, data from anatomopathological and epizootic studies contribute to the broadening of knowledge in the field of waterfowl diseases.

Further research is needed in order to explain the epidemiology of the GRV infection of geese in Poland—one of the biggest producers of goose meat.

## Data Availability

Not applicable.
